# Potential application of the probiotic *Bacillus licheniformis* as an adjuvant in the treatment of diseases in humans and animals: A systematic review

**DOI:** 10.3389/fmicb.2022.993451

**Published:** 2022-09-26

**Authors:** Hugo Ramirez-Olea, Bernardo Reyes-Ballesteros, Rocio Alejandra Chavez-Santoscoy

**Affiliations:** ^1^Tecnologico de Monterrey, Escuela de Ingenieria y Ciencias, Campus Monterrey, Monterrey, NL, Mexico; ^2^Tecnologico de Monterrey, Escuela de Ingenieria y Ciencias, Campus Estado de México, Ciudad López Mateos, MX, Mexico

**Keywords:** *Bacillus licheniformis*, functional ingredients, adjuvant in treatment, human disease, animal disease

## Abstract

The use of *Bacillus licheniformis* as a probiotic has increased significantly in recent years. Published reports demonstrate that it provides multiple benefits for health. Although there are already studies in humans and is marketed, it is mostly used in the veterinary industry still. However, its benefits could be extrapolated to humans in future. This review addresses the application of *B. licheniformis*, its sporulation, mechanisms of action, and its role in the resolution, treatment, and prevention of different conditions and diseases. It focuses on scientific advances from 2016 to mid-2022 and emphasizes the most common diseases in the general population. Most of the 70% of published studies about the health benefits of *B. licheniformis* have been published from 2016 until now. The intake of *B. licheniformis* has been related to the effects of modulation of the intestinal microbiota, antimicrobial activity, growth promotion, anti-inflammatory and immunostimulatory effects, promotion of the regulation of the lipid profile, increase of neurotransmitters, and stress reduction, among others. These results provide novel possible applications of this and other probiotics in general. Although many benefits can be reported on a microorganism, the combination with others could provide a better effect. Further studies like this need to be done to understand the specific advantages of each probiotic and its strains and therefore achieve a better selection of them for a specific disease or disorder.

## Graphical Abstract

Mechanisms of action reported for *B. licheniformis*. More than 70% of studies about the effect of *B. licheniformis* on health have been published from 2016 to 2022. The most published health effects are supported in gastric and liver diseases, diabetes, and obesity. It has also been reported for its immunological, antimicrobial, anti-inflammatory effects. 
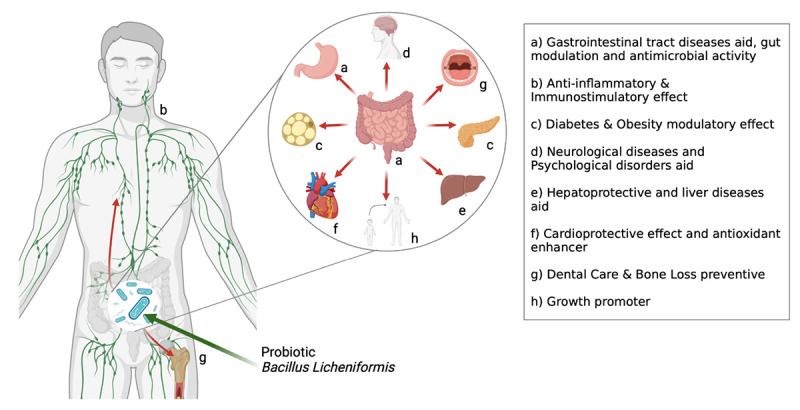

## Introduction

Probiotics are defined as “live microorganisms which when administered in adequate amounts confer a health benefit on the host” according to the Food and Agriculture Organization (FAO) of the United Nations and the World Health Organization (WHO) (Bielecka, 2006). Probiotics are also employed frequently to maintain the balance of the internal microbiota and therefore human health (Sanders et al., 2011). Recently, probiotic application is focused on reducing the risk of developing a range of illnesses since gut microbial populations are not permanent and can be altered by numerous factors such as lifestyle, diet, and antibiotics. A variety of research has established the positive effects of probiotics and their link with intestinal disorders. However, there is still information that could delve into diseases apart from gastrointestinal disorders, which can be concentrated directly or indirectly.

The gut microbiota is regulated by several factors that have also been related to disease prevention and treatment, for example, age, since it modifies proportions of microbial phylum during the different stages of life (Nagpal et al., 2018). Furthermore, dysbiosis of the intestinal microbiota has been linked to the development of disorders. A homeostatic gut microbiota population is required for the host and the microbiome to coexist in symbiotic association (Kim et al., 2019).

*Bacillus Licheniformis* is a gram-positive, endospore-forming, mesophilic bacterium belonging to the species of firmicute in the family *Bacillaceae* (Makowski et al., 2021). It can be found in raw milk as a contaminant, it is ubiquitous in soil and food on farms (Banykó and Vyletělová, 2009). It has also been isolated from buttermilk powder, pea, or mushroom soups, and in general in food as spoiling bacteria (Krawczyk et al., 2016).

*Bacillus Licheniformis* plays an important role in the biotechnology field as a strain for expression platform, compound producer, environmental applicant, and finally as a probiotic (Muras et al., 2021). This latter application has included products for human health, veterinary application, and aquaculture, alone or combined with other probiotic strains (Muras et al., 2021). Different species of *Bacillus licheniformis* probiotics have been shown and analyzed to adapt to the human gastrointestinal tract. However, some probiotics carrying *B. licheniformis*, moreover, remain considered unsafe due to their antibiotic resistance and the possibility of spreading resistance to other pathogenic bacteria. As a result, before using a strain in a procedure, it is essential to check for antibiotic resistance genes (Sorokulova et al., 2008; Muras et al., 2021).

To the best of our knowledge, an exhaustive review of *Bacillus licheniformis* focused on its uses as a probiotic, spore formation, mechanism of action, and experimental results centered on different diseases is still lacking in the literature. Therefore, this review provides an exhaust summary of the recent literature and our analysis of the data provided on the current state of knowledge about experimental and clinical research that may allow a more comprehensive perspective of the therapeutic potential of *B. licheniformis* alone or in combination.

## Bacillus spore formation and germination

Under environmental stress, such as nutritional restriction, *Bacillus spp*. bacteria produce spores (Todorov et al., 2022). Spores are a specific cell type made up of metabolically inactive cells that can withstand chemical and physical challenges like air drying, high temperatures, high pressure, UV light, and acidity. The presence of numerous distinct layers and the spore core’s high dehydration level contributes to this resistance. *Bacillus spp*. begin sporulation near the conclusion of the exponential–stationary growth phases, when nutrients are limited, and the formation of heat-resistant spores requires around 8 h (Elisashvili et al., 2019). Sporulation can be caused by nutritional stress and by exposing the cells to harsh environmental conditions such as pH and temperature extremes. This life cycle event represents a great opportunity for increasing probiotic strain development, storage, and distribution to customers from a biotechnological standpoint (Mingmongkolchai and Panbangred, 2018).

However, although these spores can potentially resist harsh conditions, there is a specific limit. For the case of *Bacillus licheniformis*, combining very high pressures and temperatures resulted in a combined impact on spore germination and inactivation and the involvement of specific germination receptors in its processes (Borch-Pedersen et al., 2017). Also, during germination, it may have specific enzymatic activities compared to other *Bacillus* species, resulting under specific nutritional optimum conditions. For example, the bacterial spore cortex is essential for spore latency and stability, and germination-specific lytic enzymes must hydrolyze it to allow proper germination and cell outgrowth (Giebel et al., 2009). The breakdown of the cortex is an important stage in spore germination. The cortex-lytic enzymes involved in the depolymerization of cortical peptidoglycan in *B. licheniformis* spores are close to those found in other *Bacillus* species; however, some investigations indicate that the primary enzymatic activity found during germination is about a lytic transglycosylase, probably SleB, and this protein seems to play a more significant role in *B. licheniformis* spore germination than in other *Bacillus* species (Aspholm et al., 2019). Each probiotic has positive characteristics on the organism depending on its specific strain and this impacts through various mechanisms (Kechagia et al., 2012). The benefits of *B. licheniformis* as a probiotic when compared to other sources of natural and encapsulated probiotics mainly lie in sporulation, since this can contribute to greater survival in the adverse conditions found in the stomach and allow its arrival to the small intestine (Casula and Cutting, 2002).

## Clinical trials of *Bacillus licheniformis*

**FIGURE 1:**
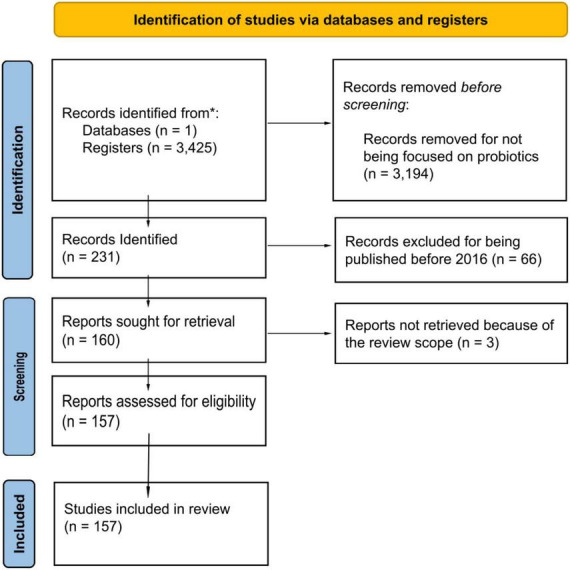
PRISMA Identification studies via database and registers. This figure illustrates the methodology followed in the development of the research on this review based on the PRISMA 2020 statement (Page et al., 2021).

The Preferred Reporting Items for Systematic Reviews and Meta-analyses guidelines were followed when conducting this review (Page et al., 2021). PubMed platform was browsed for “*Bacillus licheniformis*” as a first approach for the number of articles of this specie and then followed by “*Bacillus Licheniformis* probiotic.” The data covered the period from 2016 to mid-2022 (July 1st). The eligibility criteria were the explicit use of *Bacillus Licheniformis* alone or in combination with other strains and focused on studies on diseases and disorders with scientific proof of their correlation with the probiotics. Experimental models (humans, animals, and *in vitro*) were considered. Some limitations include the possible articles that are not on this database and the studies before 2016. However, the aim of this review is to provide a general perspective of the current state of the art. Figure 1 shows the PRISMA identification of studies via database and registers. A total of 157 articles were reviewed and represent almost 70% of the articles focused on BL as a probiotic since 1994 on this search engine. Except for 2020, which may have been mowed due to the COVID-19 outrage, there has been a noticeable increase in the number of articles since 2016.

**FIGURE 2:**
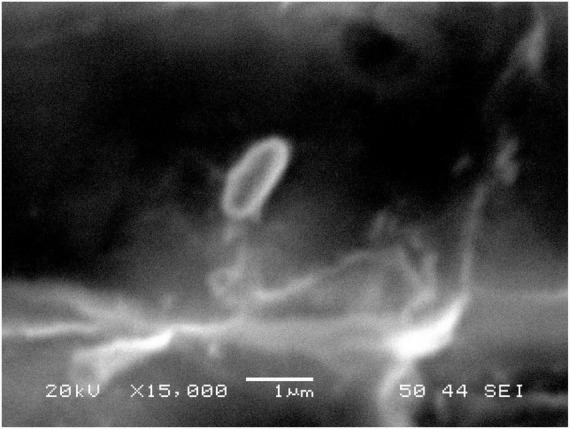
Scanning Electron Microscopy of Isolated *Bacillus Licheniformis*. The photography was taken by a JEOL JSM-6360LV using a magnification of 15,000X and an accelerating voltage of 20 kV.

**FIGURE 3:**
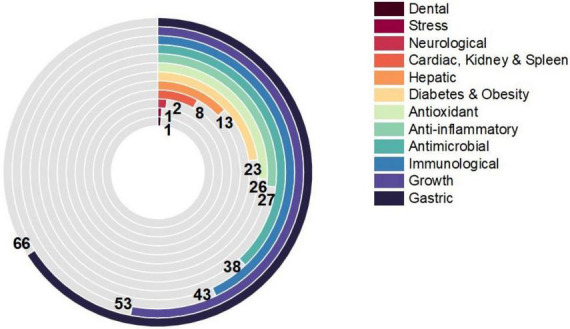
Proportion of reviewed articles on the evaluation of BL in 12 disorders. The number of articles that evaluated the effect of *Bacillus licheniformis* alone or in combination for each of the diseases is shown, and the appearance order is descending.

**FIGURE 4:**
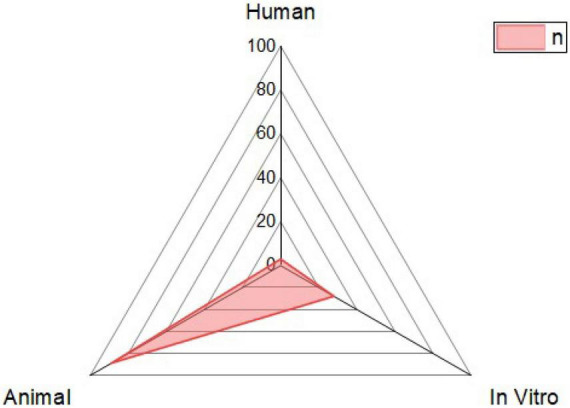
Distribution of the type of model used in the tests. It is shown if the model used to evaluate the effect of *B. licheniformis* was human, study in cells (*in vitro*), or animal models were used.

There has been a surge in interest in using probiotic supplements as mediators in health and disease in recent years. This appeal is primarily motivated by mounting the evidence of the relationship between microbiota and disease pathophysiology within the human body (Day et al., 2019). For the case of *B. licheniformis* (Figure 2), this is not an exception, its spore-forming advantages mentioned before, and the harmful conditions that can resist making it a more interesting and novel probiotic against the different diseases studied. As shown in Figure 3, its potential effect on 12 different groups of diseases and disorders was studied. The proportion of scientific articles published revealed the completeness that this probiotic could give parting from general homeostasis to a specific organ or disease. Although many of the articles combine it with other probiotic strains, its lone effect has also been deepened. Most of the studies include animal trials or *in vivo* models, almost 75% of the total were found. The distribution of studies is shown in Figure 4, and this proportion could be because of the ease to deepen results in animals compared to human models, or for being more exhaustive than the *in vitro* studies. Human models have limitations like difficulty in interpreting or generalizing the results, since the population investigated differs significantly from the one treated in daily life; also, participation in a study may have an impact on the outcome, since the restricted viewpoint of many trials leaves out critical information linked to the repercussions of the therapy on life quality, contentment, or expenses (Collet, 2000). The human trials of this review and their main outcomes are shown in Table 1. Most of the *in vitro* studies are a complement to animal studies, which may include *in vitro* biochemical assays or resected tissues. Herein, the results are shown by the group of diseases.

**TABLE 1:** Human articles summary.

Assay	Model	Relevant information	Year	Reference
Triglyceride level lowering	Human	Twelve-week trial using *Bacillus licheniformis* in combination with another spore forming bacilli probiotics have remarkable triglyceride lowering	2020	Campbell et al., 2020
Radiotherapy side effects protection	Human	In pediatric patients with central nervous system tumor, probiotics may have a critical preventive function in the etiology of gastrointestinal symptoms caused by radiotherapy. Gut barrier function, innate immunity, and intestinal repairmen can all be influenced by *Bacillus licheniformis* probiotics. Probiotics given prophylactically during irradiation therapy for CNS tumor patients can alleviate RT-related symptoms and improve cancer patients’ quality of life, in part by lowering inflammatory reactions and gastrointestinal toxicity.	2017	Du et al., 2018
Endotoxemia, Triglyceride lowering and inflammatory biomarkers	Human	After 30 days of *B. licheniformis* with other spore-based probiotic supplementation, dietary endotoxin, triglycerides, and possibly systemic inflammation were reduced. Changes in the gut microbiome, gut permeability, or a combination of the two could be the underlying cause of the observed reductions in post-prandial endotoxemia. More research is needed to confirm if a longer period of treatment with a spore-based probiotic leads to further health benefits.	2017	McFarlin et al., 2017

Related to human clinical trials of B. licheniformis, assay focuses, and main outcomes.

### Gastrointestinal tract diseases

The most addressed area in the study of probiotics is the gastrointestinal system since they proliferate in it. Therefore, many benefits have been demonstrated. For this section, a total of 68 articles showed different uses for the gastrointestinal tract when administrated *B. licheniformis*, mainly findings on microbiota modulation, followed by approaches to gastrointestinal benefits in livestock animals and finally in specific diseases such as enteritis, colitis, diarrhea, etc. One of the most relevant studies showed that in combination with *B. subtilis* could achieve an increased villus height in the ileum and a decrease in the crypt depth in the jejunum as well as the ratio of both, which can improve nutrient absorption and general digestion (Wang et al., 2021). Another study using the same combination of probiotics found that they secrete the enzymes protease, lactase, lipase, and amylase, which also provide benefits in digestion (Yang et al., 2021). Moreover, working individually with the diversity of the microbiota, an increase in *Lactobacillus* and *Firmicutes* was obtained (Chen and Yu, 2020). Also when induced colitis in rats, it lowered parameters of inflammation, weight loss, severity, and colon shortening (Li et al., 2019).

From the revised study models, the various types of studies focus on birds, mainly broilers, principally hens, and chickens, followed by those derived from pigs and marine animals, and finally rodents. Therefore, we can visualize that current gastrointestinal studies of *B. licheniformis* are more focused on the benefits for farm animals, and they seek to avoid the consumption of antibiotics by replacing them with probiotics. However, with the results on inflammation and gut microbiota regulation, it should be considered in further studies like irritable bowel disease, constipation, and colorectal diseases in animal models and humans.

### Growth promoter

Probiotics have already been proven to enhance the absorption of some nutrients (calcium, zinc, and vitamin B12) and lower the incidence of anemia, which may help children grow by preventing infections and micronutrient shortages. Previous research has explored the effects of probiotics on the diet in terms of weight and height gain in malnourished children, as well as the possibility of weight gain in well-nourished children in underdeveloped nations (Onubi et al., 2015). It has been proposed that supplementing locally accessible foods with probiotics could be a useful intervention for improving child growth, particularly in underdeveloped nations (Onubi et al., 2015).

However, for the specific application of *B. licheniformis* in animals and growth, a total of 55 studies were found, being one of the most relevant uses for this probiotic. Our study observed that most animal publications reported positive results on growth, mass gain, and feed conversion against weight gain. These publications also show that the number of viable units of *B. licheniformis* and its markers are associated with overall homeostasis. It was found that supplementing broilers with this probiotic significantly improved their weight and gain (Rodrigues et al., 2020), as well as their average daily feed intake. The growth was also associated with *B. licheniformis* competitive growth against pathogens (Chen and Yu, 2020). In lambs, the supplementation shows a significantly low feed efficiency (dry matter intake/average daily gain) (Jia et al., 2018). Also, results from a study on the pathogenicity of GFP-tagged *Vibrio parahaemolyticus* Dahv2 and the protective impact of the probiotic strain, *Bacillus licheniformis* Dahb1, on Asian catfish indicated that these organisms could be employed to manage aquatic illnesses and benefit the aquaculture sector (Gobi et al., 2016). This can potentially contribute to improving not only the health and fitness of animals intended for human consumption but also to human studies and child growth.

### Anti-inflammatory and immunostimulatory effects

Regarding the close relationship that the immune system and its inflammatory process have with the digestive system, a total of 44 studies for immune response parameters and trials were found and 27 studies had different inflammatory markers on different models. Current information showed that in combination with *B. subtilis*, a slight increase in IL-10 can be obtained, a decrease in TNF-α, and a protective effect when exposed to a specific antigen (Deng et al., 2017). Also, it has been reported that the intake of *B. licheniformis* is related to a reduction of pro-inflammatory cytokine IL-8 and an increase in of IgM and, IgG (Sun et al., 2021), while IgA antibodies, and higher concentrations of total serum proteins and globulins was also found (Gong et al., 2018). In a report about the challenge of the pathogen *E. coli* and in combination with *B. subtilis* in pigs, the authors found upregulation in the expression of TLR4, NOD2, iNOS, IL-8, and IL-22, CCL28 chemokines and its CCR10 receptor mRNA genes, and an increase of subpopulations of CD4-CD8- T-cells and no changes in the number of IL-7Rα-expressing cells (Yang et al., 2016). A specific summary of the most relevant articles on the anti-inflammatory effect of *Bacillus licheniformis* is shown in Table 2. In the aquaculture field, the enhancement of immunity is frequently mentioned as a reason why probiotics employed in the fishery are efficient at mediating protection against pathogenic diseases. Long-term feeding could maintain the persistent activation of immune cells throughout the feeding time. An application of BL probiotics was successful in sustaining systemic and mucosal immunity, as well as resistance to *A. hydrophila* (Gobi et al., 2018).

**TABLE 2:** Anti-inflammatory main articles.

BL strain/brand	Model	Relevant information	Year	Reference
*Bacillus licheniformis* Zhengchangsheng^®^ (BL20386)	Rats	A significant decrease in serum LPS level in the BL & Prebiotic group compared with the high-fat diet group, implying the alleviation of endotoxemia and systemic chronic inflammation. Rat liver was also evaluated for the levels of LPS after treatment. The liver LPS was significantly lower in the BL group compared to the HF group.	2020	Li et al., 2020
MegaSporeBiotic™	Rats	Pretreatment with probiotics BL with other spores resulted in a significant reduction in serum AST, ALT, proinflammatory cytokines (TNF-α, IL-1β), ZO-1 and TAC, as well as hepatocyte necrosis, which was similar to the well-known hepatoprotective agent, silymarin.	2020	Neag et al., 2020
*Bacillus licheniformis* Zhengchangsheng^®^ (BL20386)	Mice	Studies on the mouse model show that Dextran Sulfate Sodium induced colitis changed the diversity of the intestinal microbial composition and diversity led to an increase of inflammation in colon, which was counteracted by BL administration.	2019	Li et al., 2019
Not specified	Human	The radiotherapy treatment in experiment group markedly upregulated the serum level of ET, CRP, and TNF-α, IL-1β, IL-6.	2018	Du et al., 2018
Not specified	Rats	The treatment of probiotics decreased systematic inflammatory responses as evidenced by the decrease of TNF-α	2017	Deng et al., 2017

Most relevant articles and outcomes of B. licheniformis supplementation in anti-inflammatory effects.

When compiling the data, it was observed that the deepest and most available studies on mediators and effectors related to the immune system are found in mice and broilers, but there are still many areas of the immune system associated with this probiotic pending to be explored, since its participation on multiple metabolic pathways could aid on immune-related diseases and disorders, principally the ones related to reported inflammatory mediators.

### Antimicrobial activity

Scientific research has centered on studying pathogenic microorganisms and developing methods to prevent and treat human illnesses for many years. Conversely, in a symbiotic connection, other bacterial species may benefit the host. Antibiotics are not only the most common method used to treat infections currently (Silva et al., 2020) but also it may affect commensal bacteria in the host. Antibiotic overuse could have negative consequences both for patients and public health, such as drug-specific adverse effects and the selection of multidrug-resistant microorganisms. Probiotic formulations with immunostimulatory effects or inter-bacterial competition between beneficial and harmful bacteria are among the many that claim to benefit human health. Probiotics have been suggested as a novel and viable technique for controlling and preventing a variety of infectious diseases in this specific topic (Yang et al., 2020). For this probiotic, a current research found that *B. licheniformis* produces proticin, an antibiotic of phosphorus-containing triene (Todorov et al., 2022).

For the antimicrobial results in *B. licheniformis*, a total of 27 studies demonstrated its potential effect. In aquaculture, a study on zebrafish (*Danio rerio*) challenged the potential probiotic *Bacillus licheniformis* protective effects and *in vitro* antagonistic activities against GFP-tagged *Vibrio parahaemolyticus*. Zebrafish infected with it had 100% death, but zebrafish treated with *B. licheniformis* experienced total survival after 30 days (Girija et al., 2018). Also, in combination with *B. Breve*, an *in vitro* study showed significant inhibition against the adhesion of the pathogenic *K. rhizophila* (Rohith and Halami, 2021) and anti-vibrio activity (Sekar et al., 2019). The crude extract reveals antiviral activities against porcine epidemic diarrhea virus in Vero cells, and lower the viral shedding in piglets (Peng et al., 2019), Also in piglets, the sodium butyrate generated by *B. licheniformis* improves *Salmonella* shedding (Barba-Vidal et al., 2017). Other studies show that this probiotic has antimicrobial proteins, and high auto- and co-aggregation capabilities against pathogenic bacteria (Pahumunto et al., 2021). In the biomedical fields, the biosynthesis of silver nanoparticles employing the probiotic *Bacillus licheniformis* may be applied to manage bacterial populations that create biofilms (Shanthi et al., 2016). Multiple studies show that intestinal eubiosis is attributable to the inhibition of pathogenic microorganisms, so the aforementioned factors can achieve a reduction in infections. Although most of the studies focus on animals, multiple studies show that probiotics from the *Bacillus* family have antimicrobial properties also in humans and therefore comparable results can be expected (Hallaj-Nezhadi et al., 2022). Despite the promise advantages of probiotics for intestinal health, there is still no agreement or standardization on delivery techniques or the use of probiotic dosage forms for antimicrobial therapy; however, *B. licheniformis*, because of its potential effect as an antimicrobial agent and its survival through the gastrointestinal tract, could be a novel strain for the research of it.

### Antioxidant capacity

Oxygen species, mainly referred to as free radicals, and oxidative stress are a matter of concern nowadays. Recent studies have reported that antioxidants are produced by probiotic strains that scavenge hydroxyl radicals and superoxide anions. Molecular pathways of diabetes, atherosclerosis, inflammatory bowel disease (IBD), and damage to the heart, brain, or transplanted organs have all been linked to oxidative stress. The most acceptable species and strains for a probiotic antioxidative intervention for a certain clinical condition must be carefully considered (Hoffmann et al., 2019).

For the specific results of *B. licheniformis*, 27 studies revealed its potential effect. In a study with fish, it was shown that in combination with *B. subtilis*, they improved the levels of glutathione s-transferase (GST), glutathione reductase (GR) (Salehi et al., 2022), and combined decrease in the T-BARS marker was obtained, which indicates an increase in antioxidant enzymes (Guardiola et al., 2017). This probiotic alone had a positive impact on antioxidant capacity in the liver, serum, and intestine in birds (Zhao et al., 2020). Another study revealed that dietary supplementation with *B. Licheniformis* Dahb1 could improve innate immune function by reducing the oxidative stress linked to ammonia accumulation in tissues and blood (Gopi et al., 2022). The study for specific probiotic strains that give the most effective prevention and mitigation of oxidative stress must be continued to produce novel products with the potential to prevent oxidative stress. Further research is required to fully understand the antioxidative capabilities of prospective probiotics. Although most of the studies focus on animals, multiple studies show that probiotics from the *Bacillus* family have antioxidant properties also in humans and therefore comparable results can be expected.

### Diabetes and obesity modulatory effect

Metabolic disorders can encompass a set of diseases that lead to different routes and mechanisms that affect many vital organs; that is why for purposes of this review we have specifically limited the metabolic section to diabetes and obesity, since other parts could address gastrointestinal, hepatic, cardiovascular, and neurological problems and are deepened on the other sections or have not been studied yet. Gut microbiome regulation and probiotic beneficial metabolic effects have been investigated in patients with type 2 diabetes mellitus. Probiotics have lower total cholesterol, triglyceride levels, CRP, inflammatory biomarkers, glucose, insulin, and blood pressure regulation. Also, they have shown an improvement in HDL levels without affecting BMI or LDL levels (Kocsis et al., 2020).

Table 3 summarizes the most relevant articles on this topic. A total of 23 studies revealed the potential effect of *B. licheniformis*, alone or in combination with other probiotics or prebiotics in parameters related to diabetes and obesity, such as glucose levels, lipidic profiles, etc. Some of the mechanisms involved with *Bacillus licheniformis* are activating the AMPK pathway and suppressing the NF-κB (Lu et al., 2021). These effects specifically for *B. licheniformis* and its potential role as a supplementary therapeutic method were demonstrated in mice with a high-fat diet induced, since they showed a reduction in body weight, while it improved glucose tolerance, obesity, and insulin resistance (Cao G. T. et al., 2019). Also in high-fat diet rats, a reduction in total cholesterol, triglyceride, LDL levels, and body weight gain was observed at the same time that strains linked to obesity were reduced in microbiota composition (Li et al., 2020). Finally in humans, a 12-week trial of BL in combination with other spore-forming bacilli probiotics showed significant triglyceride reduction in patients with hypertriglyceridemia (Campbell et al., 2020). Further studies on long-term administration need to be done to complement the effect on this type of metabolic disease.

**TABLE 3:** Diabetes and obesity main articles.

BL strain/brand	Model	Relevant information	Year	Reference
*Bacillus licheniformis* Zhengchangsheng^®^ (BL20386)	Mice	Decreased weight gain, fat formation, serum lipid profiles, and proinflammatory cytokine values. Improved lipid and glucose metabolism. Nuclear factor-B activation was inhibited, phosphorylated AMP-activated protein kinase activity was enhanced in the liver, and the expression of genes involved in lipid metabolism was modulated.	2021	Lu et al., 2021
*Bacillus licheniformis* N17-02	Vitro	When compared eight different strains of *Bacillus*, *Bacillales* and *Lactobacillus*; though all had different cholesterol-removal abilities, *Bacillus licheniformis* N17-02 had the best result and presence of bile salt hydrolase gene, as well as most beneficial probiotic characteristics. As a result, it might be a suitable hypocholesterolemic probiotic candidate.	2021	He et al., 2021
Not specified	Humans	Twelve-week trial using *Bacillus licheniformis* in combination with other spore forming bacilli probiotics have remarkable triglyceride lowering.	2020	Campbell et al., 2020
*Bacillus licheniformis* YB9	Mice	Deoxynivalenol could be degraded by BL (YB9), which also had a high survival rate. Supplementing with *Bacillus Licheniformis* prevented or reduced the harm. BL could be employed as a potential probiotic supplement for increasing food and feed safety by regulating the intestinal microbiota of both animals and humans, as well as repairing intestinal dysbiosis.	2020	Wang et al., 2020
*Bacillus licheniformis* Zhengchangsheng^®^ (BL20386)	Rats	Combining *Bacillus licheniformis* with Xylooligosaccharides could be a dietary approach to alleviate gut dysbiosis, improve inflammatory status, and thereby reduce disorders linked with high fat diet obesity.	2020	Li et al., 2020
*Bacillus licheniformis* KT921419	Vitro	For 8 chosen bacterial strains from traditional fermented brine mango pickle, antioxidative, antidiabetic, and antityrosinase properties were investigated. *Bacillus licheniformis* KT921419 strain showed one of the best results on *in vitro* experiments and might be used as a new starter or auxiliary culture in a food system to impart health benefits.	2019	Ragul et al., 2020
Not specified	Mice	Without affecting food intake, *B. licheniformis* or a mixture of *Bacillus* stains reduced final body weight, improved glucose intolerance, and minimized hepatic fat accumulation in mice. Furthermore, the colonic microbiota of the *Bacillus*-supplemented and high-fat diet-fed mice differed dramatically. Probiotics derived from *B. licheniformis* could be effective in the management of a variety of metabolic disorders.	2019	Cao G. T. et al., 2019
*Bacillus licheniformis* MCC2512	Rats	The probiotics *B. flexus* MCC2427 and *B. licheniformis* MCC2512 had no negative effects on the health or behavior of the rats. Additional benefits of probiotic cultures include normal hematological parameters, lower blood cholesterol, enhanced HDL-cholesterol, increased cholic acid excretion in the stool, and higher Polyunsaturated fatty acids content in the liver. *Bacillus* bacteria in the feces was increased, whereas harmful bacteria were decreased. Overall, these probiotic cultures studied are safe and effective, and that they are likely to be safe for human ingestion.	2018	Shobharani et al., 2019
*Bacillus licheniformis* PUFSTP35	Vitro	When compared eight different strains of *Bacillus*, the most promising candidate for use as a helpful probiotic appears to be *B. licheniformis* PUFSTP35 from fermented mango pickle. *In vivo* investigations to confirm the probiotic potential of the tested isolates are required.	2017	Ragul et al., 2017

Most relevant articles and outcomes of B. licheniformis supplementation in diabetes and obesity modulation.

### Liver diseases

The gut–liver axis in most liver diseases has been proved, from the simple pathogenesis of fatty liver diseases (both alcoholic and non-alcoholic) to liver failure and, finally cirrhosis (Wiest et al., 2017). In *Bacillus licheniformis* specifically, a total of 13 studies showed its potential effect to prevent liver damage. Some of the results include the modulation of the expression of genes linked to fatty acid production and oxidation in the liver (Zhao et al., 2020), prevention of mild fibrosis and piecemeal necrosis in the liver (Wang et al., 2020), acute liver toxicity induced with acetaminophen, one of the most used analgesics and antipyretic agents in the world (Neag et al., 2020), reduction of liver weight, hepatic steatosis and effective alleviation of liver inflammation, possibly by modulating the NF-κB signaling pathway (Lu et al., 2021), and many others.

In the most relevant articles, BL interaction with gut bacteria showed a positive influence on liver damage, and a study on sheep and lambs has resulted in a significant decrease in serum levels of total bilirubin and cholesterol, parameters that point out a boost in transference from liver to bile, and leading indirectly to an improvement in liver function (Devyatkin et al., 2021). Also, the interaction of *B. flexus* and *B. licheniformis* showed a reduction in serum cholesterol, and improve in HDL-cholesterol, respectively, along with other biochemical parameters and microbiota studies that indirectly validate its efficacy and propose its use for human consumption (Shobharani et al., 2019). Another study revealed the role of BL in the homeostasis of gut microbiota and the modulation of bile acid; and in combination with *Lactobacillus salivarius* and *Pediococcus pentosaceus*, it prevented liver fibrosis and downregulated the hepatic expression of profibrogenic genes in rats (Shi et al., 2017). These results demonstrate one of the most relevant applications of *B. licheniformis* and could lead to novel applications in human hepatic diseases, both alcoholic and non-alcoholic.

### Cardioprotective effect

The microbiota in humans has been recognized as a new prospective risk factor for cardiovascular diseases. Atherosclerosis, heart failure risks, and influence of the gut microbiota in them have been previously reported. Even though animal research has revealed that gut microorganisms may influence heart disease risk, no such relation has been observed in humans (Forkosh and Ilan, 2019). For the case of *Bacillus licheniformis*, even though animal studies have not reported benefits on specific cardiovascular diseases yet, results obtained in eight studies demonstrate that not only significant improvement in hematological parameters, in general, could be achieved in combination with other *Bacillus* species (Adorian et al., 2019) but also regulation of other disorders that indirectly could be related to cardiocirculatory problems, such as triglycerides regulation (Campbell et al., 2020) for atherosclerosis, and its role in risk reduction of heart attacks, coronary diseases, cardiopathies, and many other heart illnesses (Peng et al., 2017). Also in humans, a study of 30-day probiotic supplementation of BL with other oral spore-based could reduce dietary endotoxemia (McFarlin et al., 2017). Even though endotoxemia is the result of a translocation of LPS into the circulation, studies revealed its link to an elevated risk of many cardiovascular diseases (Moludi et al., 2020).

Another application in heart failure prevention could be *Bacillus licheniformis* potential role in microbiota regulation. A recent study linked microcirculatory abnormalities in heart failure patients with anatomical and functional alterations in the gut. Emerging data suggest that gut bacteria may play a role in the pathogenesis of heart failure (Kamo et al., 2017). The breach in the intestinal epithelial barrier might allow microbial compounds to enter the bloodstream, exacerbating this disease by triggering inflammatory responses, an effect this probiotic has previously been reported to prevent in many articles and further elaborated upon. When looking for up-to-date information on cardiovascular disease, it is important to focus on the microbiota as a pathway for treatment of heart failure and other diseases. Additional research is necessary; however, the notion of the heart–gut axis might pave the way for advances in the development of novel diagnostics and therapy techniques focused on cardiovascular health.

### Neurological diseases

The gastrointestinal physiology, including digestion and gut bacteria composition, is influenced by abnormal brain activities. The gut microbiota has a strong robust bidirectional interaction with the central nervous system and impacts its outcome and mechanism of it. According to several neurological findings on the gut–brain axis, this enhances gut homeostasis. The mechanisms underlying this axis are diverse, with multiple routes involved both directly and indirectly (Suganya and Koo, 2020). Probiotic supplementation as an aid for biochemical signaling of the microbiota–gut–brain pathway, in which the intestinal microbiota, enteric nervous system, and central nervous system get connected, could have a positive influence against dysbiosis and enhancement of neuroactive substances such as serotonin or dopamine. New terms such as psychobiotics, also known as live biotherapeutics or substances with bacterially mediated beneficial effects on the brain, are currently being studied as a single or combination therapy for psychiatric and neurodevelopmental disorders, as well as possibly neurodegenerative diseases, as they could become novel treatment alternatives toward the prevention and control of brain disorders (Long-Smith et al., 2020).

Although it was found that only two studies for the specific application of *B. licheniformis* in neurological effects (apart from the psychological mentioned later), this could be mainly because of the recent attention given to this field. A 28-day trial in weaning piglets showed beneficial effects of neurotransmitters in serum and hypothalamus, serum γ-aminobutyric acid, and higher colonic concentrations of butyrate and valerate in combined probiotic supplementation (BL, *B. subtilis*, and *Clostridium butyricum*) in comparison with control and antibiotic-treated groups (Cao G. et al., 2019). An application in humans in the neurological field involved pediatric central system tumor’s side effects study caused by radiotherapy including mouth ulcer, nausea, vomiting, abdominal pain, and diarrhea. Although the effect of BL preparation on children’s survival rates and tumor recurrence was not evaluated in this investigation, it showed an improvement in intestinal function and repairment, inflammatory responses, and immunity that could lead to a better efficacy in the final treatment (Du et al., 2018). These results encourage to improve further investigations of this probiotic in neurological diseases, such as Alzheimer’s, Parkinson’s, multiple and amyotrophic lateral sclerosis, etc.

### Psychological disorders

Probiotics have been used recently in investigations to assist in negative emotions, altered behaviors, cognitive performance, and stress relief. Many scientific investigations are underway to see if probiotic supplements might assist those who are suffering from psychological stress. For *Bacillus licheniformis*, a total of two studies were found, one focused on aquaculture and the other on rats. For the purpose of this review, psychological disorders have been separated from the rest of neurological diseases to delve deeper into them.

The first one revealed that in combination with *B. Amyloliquefaciens*, results showed an improvement in larval fish survival and transport stress resistance (Tarnecki et al., 2019). One of the primary goals of any aqua farmer or entrepreneur seeking to maximize output is to reduce stress on farmed fish. This major challenge in aquaculture has prompted extensive research on reducing or eliminating the impact of stress on cultivated animals. Among the several stress reduction treatments used in aquaculture, dietary probiotic interventions have emerged as promising, empirical, and long-term solution (Ciji and Akhtar, 2021).

The second one, which focused on *B. licheniformis* alone exhibited, an improvement in behavioral changes, nervous system metabolites, neurotransmitters, and gut microbiota changes in the rat model, and demonstrated a possible new mechanism of subhealth status alleviation in psychology and behaviors, specifically because of the gut microbiome that could consume more propionic acid, resulting in alterations in brain neurotransmitters as glutamic acid (Glu), γ-aminobutyric acid (GABA), and 5-hydroxytryptophan (5-HT). At the same time, it could contribute to the reduction of norepinephrine in the brain, corticosterone, and TNF-α in the blood, as well as the inhibition of hyperactivity on the hypothalamic–pituitary–adrenal (HPA) axis and lead to anxiety reduction (Feng et al., 2022). These investigations provide new possibilities for further research on emotional disorders, their pathogenesis, and the development of their therapeutic approaches in animals and humans.

### Dental care and bone health

Periodontal healthcare and oral cavity mechanisms seem to be far from the application of probiotics interaction with the host; however, some of the topics mentioned before such as the stimulation of immune responses, inhibition of pathogens in the gastrointestinal tract, and synthesis of antimicrobial compounds could aid on the prevention and treatment of dental care diseases. In the context of disease pathogenesis, the microbiological relationship between these two mucosal locations may be linked. Several studies have found that oral bacteria can travel to the gastrointestinal tract via hematogenous and enteral axis (Kitamoto et al., 2020). It has been postulated that an oral–gut communication axis exists, but its role in the development of neurodegenerative illnesses has yet to be discovered. However, the use of probiotics for the control of various oral health disorders, like dental caries, periodontitis, gingivitis, halitosis, burning mouth syndrome, and oral cancer has been previously studied on many probiotic strains (Mishra et al., 2020). For *Bacillus licheniformis* and dental care, only one study has been reported focused on periodontitis.

Periodontitis has been linked in a lot of research to other chronic non-communicable diseases such as cardiovascular and neurological diseases (Sansores-España et al., 2021). Both deepened the beneficial effect that *B. licheniformis* has on them. In this specific study, the combined effect of BL with *B. subtillis* in rats with an experimental periodontitis-induced model was done. The main results showed a reduction in alveolar bone loss and the number of peripheral blood eosinophils in probiotic therapy concluding that with this study, further research on human clinical trials could be applied (Messora et al., 2016). This study opens new perspectives of *B. licheniformis* potential effect, not only on the whole oral healthcare applications mentioned before but also on the ones related to bone-loss diseases such as osteoporosis, a disease which has also been suggested to be approached with probiotics (Collins et al., 2018).

## Safety of *Bacillus licheniformis*

The safety of this probiotic has been tested in animal and human studies, and it can even be found over the counter as a supplement. From a study by PCR and ELISA to search for enterotoxin genes and molecules directly, none of those analyzed were found. In addition, when evaluated in BALB/c mice, rabbits, and pigs, there were no significant changes, at the histopathological, behavioral, or hematic level with chronic consumption (Sorokulova et al., 2008). The absence of genotoxicity with chronic consumption is also reported from a micronucleus assay in mice. When used topically and ophthalmic, no redness or edema was observed (Nithya et al., 2012). Some infections caused by this bacterium have been described; however, they are related to a previous lesion in tissues and/or organs, so their use in healthy patients can be considered safe (de Boer et al., 1994). The absence of antibiotic resistance in humans and animals is also observed (EFSA Panel on Additives and Products or Substances used in Animal Feed [FEEDAP] et al., 2019).

## Concluding remarks

The use of probiotics in the medical and veterinary environment has increased. Although information updates over time, the benefits of the consumption of probiotics cannot be refuted. In the case of *Bacillus licheniformis*, at this moment, most of their studies are focused on animal tests in comparison to humans. *B. licheniformis* has proven to be a probiotic for safe consumption with the ability to resist the conditions of the entire gastrointestinal system since it is an organism that has the ability to form spores and this, in turn, benefits its industrialization and handling in less than the optimal conditions for its production, getting better proliferation without losing its vitality.

Multiple benefits are observed in various pathologies and their prevention is mainly focused on the gastrointestinal and immune systems, in which the pursuit of a substitute and therapy for antibiotics after they have been used or even to replace them, stands out. In addition to the modulation of the immune response, it was found antimicrobial properties, enzyme secretion improvement, and the enhancement of eubiosis by improving the diversity in the microbiota. These benefits correlate with the improvement of diseases such as enteritis, colitis, infectious diarrhea, etc. Also, benefits associated with the circulatory system are shown, such as the modulation of markers associated with diabetes, CRP, glucose, insulin, lipid profile, and blood pressure regulation, etc. It was shown that this probiotic is associated with hepatoprotection and cardioprotection since it shows reduced dietary endotoxemia and modulation of liver toxicity and other molecules with a direct or indirect relation.

A few studies focused on Neurological and Psychological disorders were obtained, in which an improvement in the modulation of serum γ-aminobutyric acid, glutamic acid, 5-HT, and higher colonic concentrations of butyrate and valerate was observed. Moreover, better stress and anxiety response and reduction of norepinephrine could lead to a promising therapy aid in the treatment of this type of disorder. Improvements in periodontitis and other diseases associated with the uptake of nutrients for bone health, besides an improvement in antioxidant enzymes, were reported.

More studies are necessary to give a more comprehensive perspective of this probiotic in each field, but still, it is remarkable that *B. licheniformis* could be exploded not only for the diseases mentioned before but also to others such as the ones related to other fields such as dermatological, endocrine, muscle and joint, respiratory, genitourinary, etc. Although it could have some benefits for each of them, it is important to consider that this probiotic, and there are many others that could enhance its effects on a specific disease more than *B. licheniformis*.

Comparing the potential effects between probiotics and species is a complicated but necessary task to ensure the best selection of them, alone or in combination. As can be observed, many of the diseases present are the result of dysbiosis and its collateral effects; understanding the cause of it. Together with the correct probiotic treatment could be a new method to modulate the signs and symptoms that determine each disease out of range, before, after, or instead of the current treatment. With this review, we trust that further studies of each probiotic and its strains are deepened to fulfill information gaps as well as promote the study in humans and therefore achieve a better selection of them for a specific disease or disorder.

## Author contributions

HR-O and RC-S devised the review article, the main conceptual ideas, proof outline, and worked on editing and reviewing of the article. HR-O and BR-B made the systematic review and wrote the first draft of the article. All authors discussed the results and contributed to the final manuscript.

## Abbreviations

BL: *Bacillus licheniformis*.
